# Challenges faced in the management of complicated Boerhaave syndrome: a tertiary care center experience

**DOI:** 10.11604/pamj.2020.36.65.23666

**Published:** 2020-06-03

**Authors:** Sakthivel Harikrishnan, Chandramohan Servarayan Murugesan, Raveena Karthikeyan, Kanagavel Manickavasagam, Balaji Singh

**Affiliations:** 1 Surgical Gastroenterology, Government Stanley Medical College and Hospital, Chennai, India,; 2 Surgical Gastroenterology, Sri Ramachandra Institute of Higher Education and Research, Chennai, India,; 3 Madras Medical College and Rajiv Gandhi Government General Hospital, Chennai, India

**Keywords:** Boerhaave syndrome, esophageal perforation, esophageal rupture, esophagectomy

## Abstract

Spontaneous esophageal perforation is rare and is associated with high morbidity and mortality. A spectrum of various surgical modalities ranging from primary surgical repair to esophagectomy is available for its management. The optimal management of patients presenting late in a hemodynamically stable condition is not clearly defined in the literature. A retrospective review of all patients with Boerhaave syndrome managed by a single surgical team in a tertiary care center between 2008 and 2019 was performed (n = 16). Eleven patients were initially managed in the medical intensive care unit (MICU) as non-esophageal cause and 5 patients were referred after failed management (conservative/endoscopic). Demographics, clinical presentation, characteristics of perforation, initial diagnosis, and treatment were analyzed. All patients were males with a mean age of 42.2 years. A history of ethanol use was present in 6 patients. The median delay in diagnosis and referral was 16 days (range: 11-40 days). The common presenting symptoms were chest pain (n=11), dyspnoea (n=10), vomiting (n=4) and cough (n=2). The perforation was directed into right, left, and bilateral pleural cavities in 6, 8, and 2 patients respectively. The location of perforation was distal esophagus except for one patient. One patient was successfully treated with conservative management. The remaining patients underwent esophagectomy as a definitive surgical procedure. There was no significant postoperative morbidity and mortality. Esophagectomy can be done as a one-stage definitive procedure for patients with Boerhaave syndrome who present late in a hemodynamically stable condition with acceptable morbidity and good long term outcome.

## Introduction

Spontaneous esophageal perforation (Boerhaave syndrome) is rare and is associated with high mortality [1]. Prompt recognition of this condition and early surgical management is associated with a good outcome. The first successful surgical management of Boerhaave syndrome was reported by N.R.Barrett in 1947 [2]. Since then, many treatment options (conservative, endoscopic, and surgical) have emerged. A spectrum of modalities ranging from primary surgical repair to more aggressive esophagectomy is available for the surgical management of esophageal perforation. However, the optimal management for Boerhaave syndrome remains controversial. In this study, we discuss the management of 16 cases of spontaneous esophageal perforation who were referred late in a stable condition and the role of esophagectomy in them.

## Methods

A retrospective review of all patients with spontaneous esophageal perforation managed by a single surgical team between 2008 and 2019 was carried out. Patients with all other causes of esophageal perforation were excluded from the study. The following data were collected: demographic details of the patients, initial diagnosis and management in the ICU as non-esophageal thoracic cause, management details of the patients treated outside as Boerhaave syndrome, delay in diagnosis and referral, patterns of presentation, characteristics of the perforation, treatment offered, surgical approach for esophagectomy, morbidity and outcomes.

## Results

**Figure 1:**
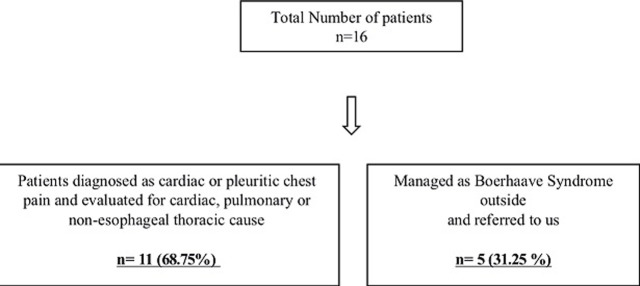
flowchart showing the number of patients treated for esophageal perforation between 2008 and 2019

A total of 16 patients were treated for Boerhaave syndrome. Eleven patients were initially managed in Medical ICU as non-esophageal thoracic cause and then referred to us after clinical suspicion of Boerhaave syndrome. Five patients were referred to us after failed surgical/endoscopic management for Boerhaave syndrome (Figure 1). All patients were males. The median delay in diagnosis was 16 days (Range: 11-40 days). The mean age was 42.4 (22-81 years). A history of alcohol use before the onset of symptoms was present in 6 (37.5%) patients (Table 1). The initial presenting symptoms were chest pain (11), dyspnoea (10), vomiting (4), and cough (2). All patients (n=16) were hemodynamically stable. Out of 11 patients admitted in MICU, 6 patients were managed as pyothorax, 3 patients as pleural effusion, and 2 patients as unstable angina. The location of esophageal perforation was lower one-third in all patients except for one patient who developed tracheoesophageal fistula after endoscopic management of lower esophageal perforation. The perforation was directed into the right, left, and both pleural cavities in 6, 8, and 2 patients respectively. Out of 16 patients with Boerhaave syndrome, one patient was successfully treated with conservative management. The remaining 15 patients underwent esophagectomy as definitive surgical management. The surgical approach was transhiatal in 8 patients, abdominal and right thoracotomy in 6 patients, and left abdomino-thoracic in 1 patient. The average length of hospital stay was 18 days. There were no significant postoperative complications and the patients were on regular follow-up. During follow-up, one patient had an anastomotic stricture and the revision of anastomosis was done by a cervical incision (Table 2).

**Table 1:** characteristics of the 16 patients with complicated Boerhaave syndrome

Men / Women	16/0
**Age characteristics**	
Mean age (years)	42.2
Range (Years)	22- 81
**Hemodynamic parameters**	
Pulse rate (Mean)	104 / min
Systolic Blood pressure (Mean)	100 mm hg
History of ethanol use	6 (37.5 %)
**Presenting symptoms**	
Chest pain	11 (68.7%)
Dyspnoea	10 (62.5%)
Vomiting	4 (25%)
Cough	2 (12.5%)
Delay in diagnosis and referral from ICU (Median)	16 days
**Number of patients initially managed for non-esophageal thoracic cause (n=11)**	
**Initial diagnosis**	Number of patients
Pyothorax	6 (54.5 %)
Pleural effusion	3 (27.2 %)
Unstable angina	2 (18.1 %)
**Location of Perforation**	
Distal	15/16*
**Perforation into the pleural cavity**	
Right	6 (37.5%)
Left	8 (50%)
Right and Left	2 (12.5%)

* One patient had a perforation in the distal esophagus which was managed by stenting-Post stenting he developed tracheoesophageal fistula.

**Table 2:** surgical management and the outcomes

Conservative	1
Surgical management	15 (93.75%)
**Surgical approach**	
Transhiatal	8 (50 %)
Abdominal + right thoracotomy	6 (37.5 %)
Left abdominothoracic	1 (6.2 %)
The average length of hospital stay (days)	18
**Morbidity**	
Surgical site infections	2
Anastomotic leak	0
Atelectasis of one or both lungs	6
Mortality	0
Anastomotic stricture *	1

* 1 patient had anastomotic stricture which was revised by a cervical incision

Management of 5 patients who were referred to us after failing surgical/nonsurgical management is discussed below:

**Case 1:** a 60-year-old male was presented with dyspnoea and right hydropneumothorax. Right ICD was inserted and then referred to us for persistent drainage of ICD. He underwent transhiatal esophagectomy.

**Figure 2:**
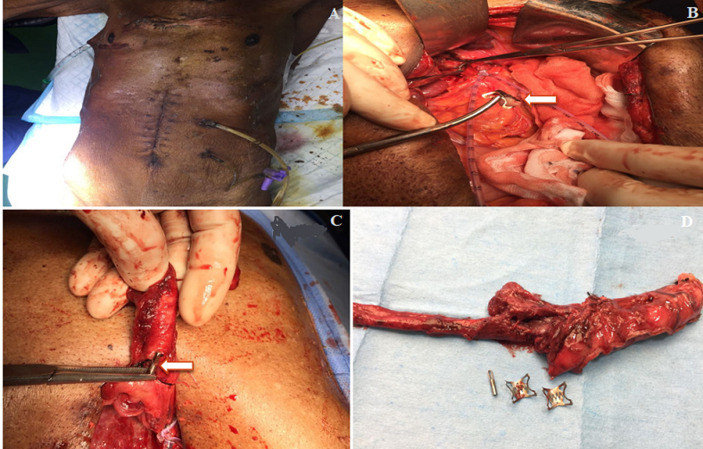
A) 81 year old patient referred with persistent leak from right ICD; B,C) picture shows right transhiatal esophagectomy specimen with hemoclip and OVESCO clip (arrow) retrieved from the esophageal rent; D) transhiatal esophagectomy specimen with the OVESCO clip and the hemoclips

**Case 2:** an 81-year-old male was diagnosed with Boerhaave syndrome. He was initially managed with ICD insertion. Transhiatal esophagectomy was attempted but failed. Hence venting gastrostomy and feeding jejunostomy was done. He was managed endoscopically by the placement of the OVESCO clip followed by hemoclip. He was then referred to us for further management because of persistent drainage (Figure 2).

**Figure 3:**
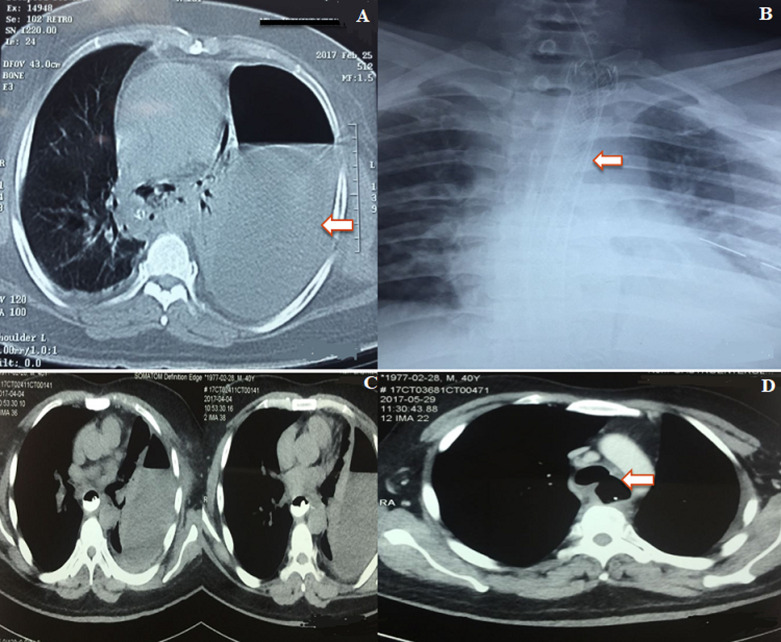
A) CT chest showing left pleural effusion (white arrow) post esophageal rupture; B) chest x-ray showing endoscopically placed SEMS in situ; C) post stent removal, CT thorax showed a 3mm esophagopleural fistula from the distal esophagus to the left pleural cavity; D) post repeat stenting and stent removal, CT thorax showed tracheoesophageal fistula (arrow) and no leak from the distal esophageal perforation

**Case 3:** a 42-year-old male with Boerhaave syndrome developed tracheoesophageal fistula after endoscopic management of the perforation. He was initially managed with left ICD insertion for left pyothorax. Post ICD insertion endoscopy showed 26 to 31 cm esophageal rent for which stenting was done. The stent was removed after a month and repeat computed tomography (CT) showed esophago-pleural fistula with 3 mm rent on the left lateral wall of the distal esophagus. Fistula closure was initially attempted by endoscopic APC and fibrin sealant followed by another session of glue, thermal coagulation, and Beta 2 SEMS deployment. Repeat endoscopy was done after a month and it showed closure of the previous fistula site but the patient developed a tracheoesophageal fistula and then he was referred to us for further management. He was managed by right thoracotomy and layered fistula closure with vascularised intercostal muscle flap and subtotal esophagectomy with retrosternal gastric pull up, esophagogastric anastomosis with feeding jejunostomy (Figure 3).

**Case 4:** a 48-year-old male was initially managed with ICD insertion for Boerhaave syndrome and then referred to us for persistent drainage. He underwent right thoracotomy and esophagectomy.

**Figure 4:**
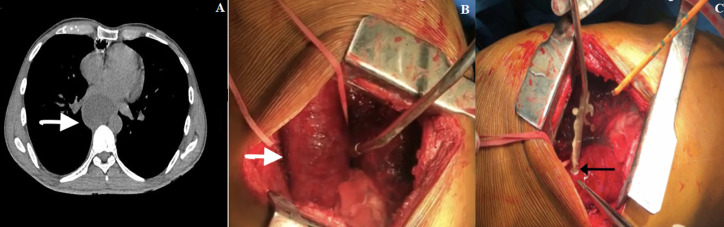
A) CT thorax showing the mucocele of the remnant esophagus (arrow); B) intraoperative picture showing giant mucocoele of the remnant esophagus looped (arrow) by a right thoracotomy incision; C) drainage of the mucocele with a suction catheter (arrow) followed by remnant esophagectomy after decompression

**Case 5:** a 33-year-old male with Boerhaave syndrome was initially managed with ICD insertion and then he underwent cervical esophagostomy and lower esophageal stapling. He was taken up for the restoration of esophageal continuity later, but because of dense adhesions, he underwent gastric pull-up. After 3 months, he had persistent hiccups and on evaluation was found to have giant mucocele of the remnant esophagus. He was referred to us for further management. He underwent right thoracotomy, mucocele drainage, and remnant esophagectomy (Figure 4).

## Discussion

In 1724, Hermann Boerhaave, a Dutch physicist, first described spontaneous rupture of the esophagus, which typically occurs after forceful emesis [3]. Spontaneous perforation of the esophagus also called Boerhaave syndrome or Barogenic rupture is a highly morbid and lethal condition. It comprises about 15% of total esophageal perforations. Spontaneous full-thickness longitudinal tear in the esophagus occurs due to a sudden increase in the intraesophageal pressure against a closed glottis. Repeated retching and emesis is the most common cause of spontaneous perforation. The perforation is usually located in the left posterolateral wall of the distal esophagus and the defect is about 3-8 cm. The main goals in the management of spontaneous esophageal perforation are: 1) resuscitation and supportive care; 2) prevention of sepsis by draining the contaminated areas (mediastinum and the pleural space) and starting broad-spectrum antibiotics; 3) sealing the perforation and maintaining the foregut continuity if possible. There is a temporal association between the esophageal perforation and the mortality rate. The reported rates of mortality are between 16% and 24% [4], but it increases by up to 50% when treatment is initiated after 24 hours [5]. The most important survival predictor is the early onset of treatment. Various treatment modalities available for esophageal perforation include conservative, endoscopic, and surgical methods. Surgical methods can either be primary surgical repair, diversion/exclusion procedures, and esophagectomy.

Endoscopic methods eliminate the morbidity and mortality associated with thoracotomy or laparotomy in a septic patient. Various endoscopic modalities for the management of perforation include hemoclip, OVESCO clip, endostitch, and SEMS placement. Stent migration is of specific concern in patients with Boerhaave syndrome as the stent has to cross the gastroesophageal junction to seal the perforation effectively. Freeman *et al*. reported endoscopic management of 19 cases of spontaneous esophageal perforation where 17 patients had occlusion of the perforation. He used Polyflex stent for all patients, oral intake was resumed within 72 hours for 14 patients (82%) and the stents were removed with no residual leak at a mean of 20±15 days. He also did additional procedures like percutaneous gastrostomy in 19 patients, video-assisted thoracoscopic decortication in 5 patients (26%), and tube thoracostomy in 4 (21%) along with endoscopic stenting [6]. Leers *et al*. used Self Expandable Metallic Stents (SEMS) (Ultraflex, microvasive) for sealing of esophageal leaks associated with spontaneous esophageal perforation, anastomotic leaks, and iatrogenic esophageal perforation. The median size of the perforation was 1.3cm (0.5-4 cm) and the majority of the cases (74%) were malignant perforations. Out of 31 patients who underwent endoscopic SEMS placement, 26 patients had sealing of the perforation [7].

Torben Glatz *et al*. analyzed the management and outcome of stents for spontaneous esophageal perforation of 16 patients. They have used covered self expanding stent for all patients. Patients who received endoscopic treatment within 48 hours had a reduced rate of treatment failure (42%) when compared to patients who received treatment beyond 48 hours (75%). Patients who underwent second stenting had a higher rate of long term esophageal stenosis and persistent dysphagia. Two patients had extensive perforation (5 cm and 10 cm) and they underwent esophagectomy after primary stent failure [8]. Hendren and Henderson described one stage esophageal resection and reconstruction for esophageal perforation in 1968 [9]. Altorjay *et al*. advocated esophagectomy for esophageal perforation of varied causes in 27 patients. They proposed that an esophagectomy is a viable option even in patients with delayed diagnosis as it eliminates the intrathoracic sepsis and the affected esophagus [10]. Orringer *et al*. reported the management of 24 patients of esophageal perforations of varied causes. They did esophagectomy with single-stage reconstruction in 13 patients and esophagectomy with delayed reconstruction in 11 patients. Esophagectomy was done by a transhiatal approach without thoracotomy in 15 (63%) patients and by a transthoracic approach in 9 patients [11]. Many studies [10-16] have proved esophagectomy as a better option for delayed esophageal perforation and are summarised in Table 3.

**Table 3:** summary of the articles discussing esophagectomy as a better option for esophageal perforation

Author	Sample size	Results	Outcome
Orringer and Stirling (1980) [11]	11`	Esophagectomy removed the source of sepsis while conservative procedures have more morbidity	The mortality rate was 13% (3 patients)
Salo *et al*. (1993) [12]	34	19 managed conservatively and 15 underwent esophagectomy	The mortality rate was 68% for the conservative procedure and 13% for esophagectomy
Iannettoni *et al*. (1997) [13]	42	26 managed conservatively and 16 underwent esophagectomy	Esophagectomy was a one-step procedure. Patients who were managed conservatively required at least one more additional intervention for persistent dysphagia
Altorjay *et al*. (1998) [10]	27	16 patients underwent esophagectomy	The complication rate was higher for conservative management than esophagectomy (25.9 vs 14.8)
Bresadola *et al*. (2008) [14]	14	6 patients underwent esophagectomy	In patients with sepsis, it demands an aggressive approach such as esophagectomy
Sutcliffe *et al*. (2009) [15]	11	6 patients managed surgically	Mortality is higher in conservative management than surgical management (75% vs 17%)
Tettey *et al*. (2011) [16]	10	3 patients managed conservatively and 7 underwent esophagectomy	The mortality rate was low in esophagectomy group (1 patient)

## Conclusion

Our series demonstrates the effectiveness of esophagectomy as a one-stage procedure for the management of Boerhaave syndrome who present late and for those patients who present after failed endoscopic therapy. All our patients were hemodynamically stable and presented more than 10 days after perforation. In patients who survive the acute insult, esophagectomy as a one-stage surgical procedure is a better alternative in terms of morbidity and mortality.

### What is known about this topic

- Early diagnosis and treatment is crucial for Boerhaave syndrome;
- Patients who present within 24 hours of perforation have the best survival;
- The management of patients who are diagnosed late (conservative/endoscopic/surgical) is unclear.

### What this study adds

- In Boerhaave syndrome, if the patients survive the acute insult, surgical esophagectomy is a better treatment option than conservative/endoscopic management;
- Transhiatal esophagectomy can be done as a one-stage definitive procedure for patients who present late in a hemodynamically stable condition with acceptable morbidity and good long term outcome.
